# Successful combination chemotherapy with irinotecan hydrochloride and cisplatin for primary gastric small cell carcinoma: report of a case

**DOI:** 10.1186/1477-7819-11-263

**Published:** 2013-10-07

**Authors:** Hitoshi Funahashi, Hirotaka Miyai, Takehiro Wakasugi, Hideyuki Ishiguro, Yoichi Matsuo, Masahiro Kimura, Hiromitsu Takeyama

**Affiliations:** 1 Department of Gastroenterological Surgery, Nagoya City University Graduate School of Medical Sciences, 1 Kawasumi, Mizuho-cho, Mizuho-ku, Nagoya 4678601, Japan

**Keywords:** Small cell carcinoma, Cisplatin, Irinotecan hydrochloride, Combination chemotherapy

## Abstract

Primary gastric small cell carcinoma is a rare and aggressive malignant disease with a poor prognosis that was first reported in 1976 by Matsusaka *et al*. The incidence is very low and the clinicopathological features are similar to those of small cell lung carcinoma.

We herein report a case of successful treatment by combination chemotherapy consisting of irinotecan hydrochloride and cisplatin for primary gastric small cell carcinoma. The patient was a 71-year-old male who was admitted to a local hospital with anemia. Gastrointestinal endoscopy revealed the presence of advanced gastric carcinoma at the upper region of the stomach. The patient underwent surgery, and the pathological diagnosis was small cell carcinoma due to the presence of the typical features of small round cells with scant cytoplasm that were positive for synaptophysin and chromogranin A in the resected specimen. The patient underwent subsequent combination chemotherapy, which provided him with over 1 year of survival and a good quality of life. We also present a review of the literature regarding chemotherapy for primary gastric small cell carcinoma.

## Background

Gastric carcinoma is a widespread malignant disease, and the worldwide mortality and incidence rates are 6.4 and 8.7 per 100,000, respectively [1]. These rates are particularly high in Japan (27.3 and 60.4 per 100,000, respectively) [2]. Gastric small cell carcinoma (GSCC) was first described as a subtype of gastric carcinoma in 1976 by Matsuzaka *et al*. [3]. Currently, GSCC is defined as one of the neuroendocrine tumors (NETs) according to the World Health Organization classification. The biological characteristics and clinicopathological features of GSCC are similar to those of small cell lung carcinoma (SCLC) [4-6], but GSCC is known to be more aggressive and malignant compared to SCLC because GSCC is more resistant to chemotherapy [6]. Effective treatment strategies for GSCC have not yet been established, because it is very rare, with an incidence of about 0.02% of all gastric carcinomas, even in Japan. We believe that surgery and subsequent systemic chemotherapy represent an effective therapeutic approach for GSCC. Several reports have recommended using the same therapeutic strategies as those used for SCLC [6,7]. Standard chemotherapy for SCLC consisted of VP-16 and cisplatin (CDDP) for a long time [8]. Recently, combination irinotecan hydrochloride (CPT-11) and CDDP chemotherapy has been shown to be more effective for SCLC based on the results of phase II and phase III clinical trials [9-11]. Therefore, we used combination CPT-11 and CDDP chemotherapy to treat the present patient.

We herein report a case with advanced GSCC that was successfully treated by surgery and subsequent combination CPT-11 and CDDP chemotherapy.

## Case presentation

**Figure 1:**
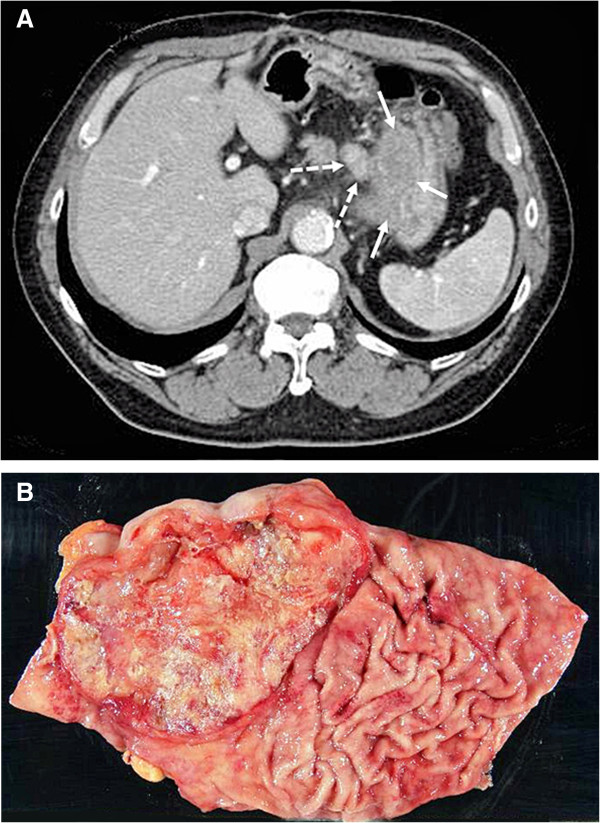
Tumor location and form **Tumor location and form.** Abdominal computed tomography (CT) scans showed carcinoma at the esophagogastric junction (white arrow) and lymph node swelling (dotted arrow) before surgery **(A)**. Macroscopic findings of the resected stomach: a Borrmann type III tumor that measured approximately 100 mm in diameter was located around the esophagogastric junction **(B)**.

A 71-year-old Japanese male went to a local hospital for postoperative follow-up of colonic carcinoma. Then anemia was noted, and gastrointestinal endoscopy revealed a large ulcerated tumor around the esophagogastric junction. Tumor biopsy specimens showed adenocarcinoma, and he was admitted to Nagoya City University Hospital for curative treatment. His family history was unremarkable, and medical history included acute myocardial infarction, bladder carcinoma, and colon carcinoma. Laboratory data were within normal limits except for anemia indicated by a hemoglobin (Hb) level of 9.4 g/dL (normal range: 13.2 g/dL < Hb < 17.2 g/dL). Serum levels of carcinoembryonic antigen (CEA) and carbohydrate antigen 19–9 (CA19-9) were also within normal limits: 1.6 ng/mL (normal range <3.5 ng/mL) and 3.7 U/mL (normal range <37 U/mL), respectively. On computed tomography (CT), the tumor appeared as a thickening of the gastric wall with metastasis to perigastric lymph nodes (LN) (Figure 1A). Re-examination with gastrointestinal endoscopy revealed a Borrmann type III tumor located around the esophagogastric junction, which was diagnosed as GSCC upon histological examination of a biopsy specimen at our hospital. After providing informed consent, and following a sufficiently detailed explanation about his disease, the patient wished to undergo surgery. Surgical resection by proximal gastrectomy was performed. While total gastrectomy is the standard treatment for such cases, proximal gastrectomy was selected during surgery, because his small bowel, which was one lump due to previous surgery, could not be elevated to allow Rou-en-Y reconstruction.

**Figure 2:**
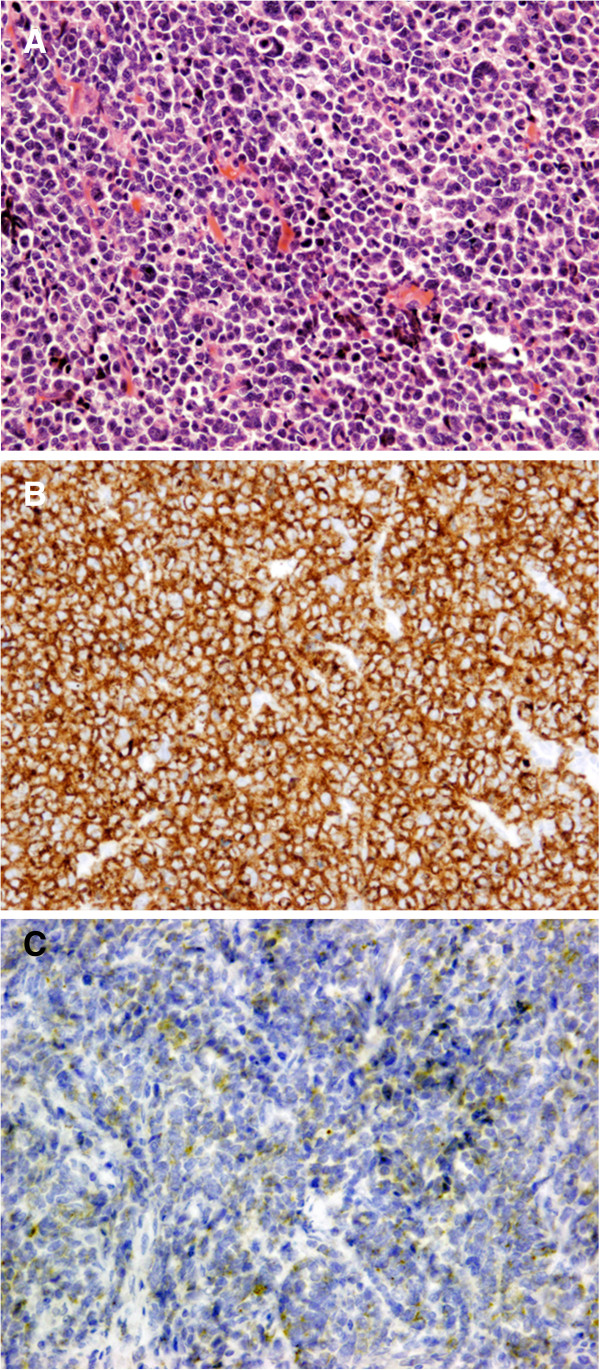
Microscopic examination of the tumor **Microscopic examination of the tumor.** Hematoxylin and eosin staining demonstrated small cells with hyperchromatic nuclei and scant cytoplasm (x200) **(A)**. The tumor cells were positive for synaptophysin **(B)** and chromogranin A (x200) **(C)**.

Resected specimens showed a Borrmann type III tumor that measured approximately 100 mm in diameter around the esophagogastric junction (Figure 1B). Histopathologic examination revealed that the tumor consisted of small cancer cells with dense chromatin nuclei (Figure 2A). Immunohistochemically, the tumor cells were positive for synaptophysin (Figure 2B) and chromogranin A (Figure 2C). The final histological diagnosis was GSCC. The pathological stage was IIIA (T3, N1, M0) according to the classification proposed by the Japanese Gastric Cancer Association [12].

**Figure 3:**
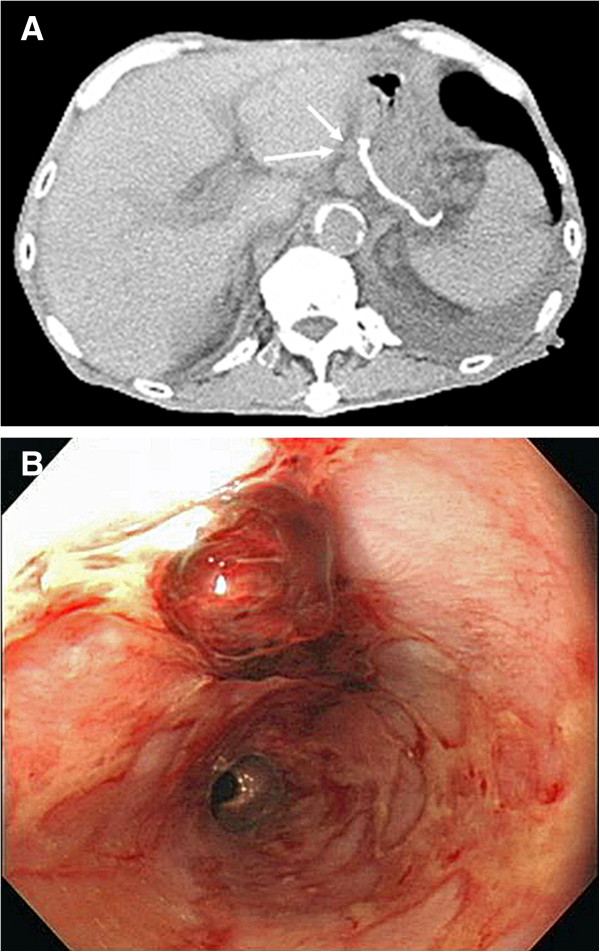
Postoperative course **Postoperative course.** Five months after the last course of chemotherapy, computed tomography (CT) scans revealed a recurrent regional lymph node (arrow) but no recurrence in the liver **(A)**. A contemporary gastrointestinal endoscopy revealed hemorrhage from an esophagus longitudinal ulcer, but no carcinoma recurrence was apparent in the esophagus **(B)**.

After obtaining informed consent from the patient, we started the systemic chemotherapy with CPT-11 and CDDP. On day 1, CPT-11 (60 mg/m^2^) was administered, followed by administration of CDDP (80 mg/m^2^) over 2 hours with adequate hydration. The same dose of CPT-11 was administered on day 15, and this regimen was repeated every 4 weeks. Although NCI-CTC grade 2 febrile neutropenia and diarrhea and grade 1 nausea were observed, these side effects were resolved following pharmacologic intervention with G-CSF and antidiarrheal medications. After three courses, the patient declined further chemotherapy due to unbearable side effects. After 12 months (4 months after chemotherapy was stopped), his CEA level increased from 1.6 ng/mL to 12 ng/mL. Cancer recurrence was confirmed in a regional lymph node on CT (Figure 3A). Although we suggested re-initiation of systemic chemotherapy, the patient declined treatment. Fourteen months after surgery, the patient died of hemorrhage due to esophageal ulcer and subsequent aspiration pneumonia (Figure 3B).

## Discussion

Small cell carcinoma is a malignant disease that is frequently observed in the lung [5]. While extrapulmonary small cell carcinoma has been reported in the gastrointestinal tract, head, neck, urinary tract, and genitals [13,14], incidence of extrapulmonary small cell carcinoma is much lower than that of SCLC. Among these cancers, primary GSCC is extremely rare. Matsusaka *et al*. first reported GSCC in 1976, and revealed that the incidence was less than 0.1% of all gastric carcinomas in Japan, even though the incidence of gastric adenocarcinoma in Japan is much higher than in other countries [3].

It is generally difficult to diagnose GSCC preoperatively. Kusayanagi *et al*. reported that only 40% of patients with GSCC were diagnosed correctly before surgery [15]. This may be due to the fact that carcinoma cells can differentiate in many aspects. Matsui *et al*. also suggested that small cell carcinomas originate from totipotent primitive cells, which can result in dual or multiple differentiation into a mixture of small neoplastic cells, squamous cells, and adenocarcinomatous cells [16]. Furthermore, tumor cells may not be detectable in biopsy specimens, because GSCC proliferates in the submucosal layer in many cases. In the present case, adenocarcinoma was first diagnosed at a local hospital. however, re-examination at our hospital enabled a correct diagnosis by cell morphology and immunohistochemical findings.

Immunohistochemical examination is well known to be valuable for the diagnosis of GSCC. GSCC is unique in its positive reactions to synaptophysin and chromogranin A, although 10-20% of GSCCs demonstrate negative reactions for these tumor markers [17]. Histologically, the features of GSCC are similar to those of SCLC: they have a scanty cytoplasm and small-sized oval nuclei with inconspicuous nucleoli [18,19]. Therefore, the final diagnosis of GSCC should be based on both morphologic features and immunohistochemical reactions. In the present patient, the final diagnosis of GSCC was achieved due to positivity for both markers and histopathologic characteristics similar to SCLC in the resected specimen.

Like SCLC, GSCC is an aggressive disease and the clinical course is generally very poor. Previous reports revealed that almost every patient with GSCC dies within 1 year [6,15], because GSCC shows a high incidence of vasculolymphatic invasion, marked deep infiltration, and distant metastases at diagnosis [16,20]. Unfortunately, there is no standard effective treatment strategy for GSCC. Various treatment modalities have been proposed including surgery, chemotherapy, radiation therapy, and various combination of the above [15,20]. Although surgery and/or adjuvant chemotherapy have been evaluated in several studies, the results have been unsatisfactory [6,21-23]. Although the effects of the treatment based on radiation therapy for esophagus or thyroid small cell carcinomas were reported [24,25], radiation therapy for GSCC has not been reported. A few reports revealed that surgical resection appeared to be effective, with survival ranging from 9 months to more than 5 years for patients with GSCC limited to the stomach [3,26,27]. In contrast, in another report, massive hepatic metastases occurred in a short period in a case with early-stage disease, and the patient died within 6 months after definitive surgery [28]. Most patients with GSCC receive combination therapy consisting of surgery and subsequent chemotherapy, similar to that used for conventional gastric cancer, which involves 5-FU-based chemotherapy [6]. Nevertheless, no standard treatment strategy has been established for GSCC due to insufficient efficacy. Furthermore, the prognosis in GSCC is poorer than that for gastric adenocarcinoma, particularly in patients with advanced-stage diseases: the reported median survival for GSCC is 7 months [6,7,16,28]. Treatment of GSCC with chemotherapy regimens used in SCLC may be more effective than other regimens. In fact, some reports have revealed that some SCLC chemotherapy regimens are also effective against GSCC [4,5,29]. The most commonly used combination chemotherapy regimen for patients with SCLC is etoposide and CDDP [8]. This regimen yields an overall response rate of 80-95% in patients with limited disease and 60-80% in those with extensive disease [30]. Recently, combination CPT-11 and CDDP chemotherapy has also been shown to be effective for SCLC. The results of a phase III trial that compared CPT-11 and CDDP with CDDP and etoposide indicated that the overall response rate in the CPT-11 and CDDP group was significantly higher than that in the CDDP and etoposide group. Moreover, the median survival rate was 12.8 months in the CPT-11 and CDDP group compared to 9.4 months in the CDDP and etoposide group [11]. Based on the results of this trial, we elected to use a combination CPT-11 and CDDP regimen in the present patient. After three cycles of treatment, the patient experienced National Cancer Institute’s Common Toxicity Criteria (NCI-CTC) grade 2 febrile neutropenia and diarrhea, and grade 1 nausea. The patient was sensitive to these side effects and refused further chemotherapy or alternate therapy. However, his duration of response and progression-free survival continued for approximately 1 year. Although previous reports revealed that almost all patients die within 6 months of being diagnosed with GSCC [6,7,15,16], this patient survived for longer than 1 year with a satisfactory quality of life. Unfortunately, he died due to hemorrhage from an esophageal ulcer and subsequent aspiration pneumonia. It was not determined whether the esophageal ulcer was due to disease recurrence. However, recurrence was not observed in the esophageal mucosa, and no recurrence was present in the liver. If he had undergone continuous chemotherapy, he may have survived for a longer time.

## Conclusions

Because the incidence of GSCC is very low, no standard treatment strategy has been established. Indeed, effective surgical treatment is needed and subsequent intensive chemotherapy should be considered to promote long-term survival. Combination chemotherapy consisting of CPT-11 and CDDP appears to be effective for the treatment of primary GSCC. Further trials and experience in additional cases are required to fully evaluate the efficacy of this CPT-11 and CDDP regimen and to help establish the best treatment strategy for patients with GSCC.

## Consent

Written informed consent was obtained from the patient’s family for the publication of this case report and any accompanying images. A copy of the written consent is available for review by the Editor-in-Chief of this journal.

## Abbreviations

CA19-9: Carbohydrate antigen 19–9; CDDP: Cisplatin; CEA: Carcinoembryonic antigen; CPT-11: Irinotecan hydrochloride; CT: Computed tomography; GSCC: Gastric small cell carcinoma; Hb: Hemoglobin; LN: Lymph nodes; NCI-CTC: National Cancer Institute’s common toxicity criteria; NETs: Neuroendocrine tumors; SCLC: Small cell lung carcinoma.

## Competing interests

Each co-author certifies that he has no commercial interest that might constitute a competing interest in connection with the submitted article.

## Authors’ contributions

HF was responsible for the writing. TW, YM, and HI participated in data collection. HM, MK, and HT participated in literature searching. All authors have read and approved the final manuscript.
